# Modeling heart rate (HR) dynamics to reconstruct missing HR data in trail running

**DOI:** 10.1186/s13102-026-01727-4

**Published:** 2026-05-07

**Authors:** Tjorven Schnack, Raimundo Sanchez, Arnold Baca

**Affiliations:** 1 Department of Sport and Human Movement Science, Centre for Sport Science and University Sports, University of Vienna, Auf Der Schmelz 6a, Vienna, 1150 Austria; 2 Vienna Doctoral School of Pharmaceutical, Nutritional and Sport Sciences, University of Vienna, Josef-Holaubek-Platz 2, Vienna, 1090 Austria; 3 School of Health and Rehabilitation Sciences, The University of Queensland, St Lucia, Brisbane, 4072 Australia

**Keywords:** Heart rate, Missing data, Reconstruction, Running, Trail, HR model, Imputation

## Abstract

**Background:**

Heart rate (HR) is widely used to guide exercise intensity because it is non-invasive and easy to measure. However, outdoor recordings often contain artifacts and gaps, which can bias downstream metrics if not reconstructed accurately. Common reconstruction methods (e.g. linear interpolation) perform well in short gaps but ignore HR kinetics and changing external load, which is problematic in trail running with frequent intensity changes. The present study presents a HR reconstruction approach that leverages GNSS data and a model of HR dynamics.

**Methods:**

12 recreational trail runners completed a total of 53 trail bouts, of which 37 were included in the final analysis (1 Hz GNSS, 130 Hz chest-strap ECG). Gaps of 1-800 consecutive heart beats were simulated at four phases representative of trail running: *onset*, *uphill*, *switch* (uphill-to-downhill), and *downhill*. Energy expenditure was estimated from GNSS data and mapped to heart rate using a first-order differential equation, with individualized parameters. The end of the reconstructed gap was aligned with post-gap measurements via a linear drift correction. HR model based reconstructions without (HRM) and with drift correction (HRMD) were compared against baseline linear interpolation (LI). Reconstruction accuracy was assessed via RMSE and a linear mixed-effects model, and bias was summarized using mean error (ME) and limits of agreement (LoA).

**Results:**

Overall, HRMD and LI had a similar low median RMSE (approx. 2 bpm), both outperforming HRM (approx. 3 bpm). However, significant interaction effects indicated condition-dependent performance. HRMD was significantly better than LI for non-steady exercise intensities at longer gaps (*onset* and *switch* for 200 missing beats or more). LI slightly outperformed HRMD during steady *uphill* (200 missing beats or more). Bias and dispersion favored HRMD: ME remained near zero with LoA less than 10 bpm across all conditions. LI showed large, length-dependent bias at *onset* (ME up to 14.6 bpm; LoA up to 20.3 bpm). This indicates that HRMD provides an absolute reduction of mean error of up to 14 bpm (at *onset* for 800 missing beats).

**Conclusions:**

LI is adequate for short gaps and steady intensity, but errors grow and become biased during long, non-steady gaps. Integrating GNSS-derived energy expenditure with individualized HR dynamics and drift correction (HRMD) reduces error and bias in these challenging conditions. Practical implementation of this reconstruction approach is accessible since wearables facilitate synchronous HR and GNSS recordings. However, it remains uncertain how the results generalize to different demographics and other running disciplines.

## Introduction

Heart rate (HR) is a commonly used proxy for exercise intensity during physical activity [1]. Unlike other indicators of exercise intensity, such as oxygen uptake or blood lactate concentration, HR can be measured non-invasively and with minimal technical effort, leading to widespread adoption in professional and amateur sports [2]. Using heart rate (HR) to guide the training intensity distribution of a training schedule can help optimize running performance [3, 4]. Moreover, metrics such as the HR recovery and HR kinetics can give insights into the training status [5–7], provided that HR recordings are accurate and complete.

Despite recent advances in wearable technologies, HR monitors are susceptible to measurement errors. Even ECG chest straps, which are considered accurate, can suffer from disturbed signal conduction, motion artefacts, displacement through motion, or incorrect placement [2, 8]. This may cause measurement artefacts or missing data [9]. Literature suggests that during intense running exercise, around 4% of recorded HR-values may be erroneous, most commonly due to missed individual beats [9]. In practice, long intervals of consecutively missing samples are not unusual. Sweat can compromise the electrical conduction between electrode and skin [8], and torso movement or ground-impact during running can displace the sensor, leading to missed beats. Especially in minimally controlled environments, like outdoor running, errors are often not immediately noticed, delaying the correction of the root cause. Other cause for long intervals of missing data can be sensor malfunctions or connectivity issues.

In literature different longitudinal imputation methods were applied to reconstruct missing HR data. The methods can be grouped into online and offline approaches. Online approaches, such as forward fill [10] and mean carried forward [10], only use values before the gap of missing data for reconstruction. Offline approaches use values before and after the gap, which makes them more accurate and preferable if real-time processing is not required. For offline HR reconstruction, previous studies employed linear interpolation [9–12], piecewise cubic Hermite interpolating polynomial [11, 13], cubic spline [9–12], and nearest neighbor [11, 13]. Linear interpolation provided good performance in multiple comparison studies [9–12]. One study suggests that different reconstruction methods may be required depending on the amount of missing data [11]; although the analysis addressed HR variability indices, similar considerations likely hold for HR itself.

The majority of the above mentioned studies took place in a health care setting [10–13]. Only one study was in the context of running, but this study focused on HR variability metrics and did not consider consecutively missing samples [9]. Further shortcomings are that the current reconstruction methods do not reflect the underlying physiological principles, and it is unclear how the reconstruction accuracy behaves over different lengths of consecutively missing data. This is an issue during physical activity where HR responds to changes in external load. For example, at the onset of running, HR typically exhibits an exponentially attenuated increase [14]. Yet, linear interpolation reconstructs missing values with straight lines. Especially for longer data gaps, this can distort peak amplitudes and onset dynamics, leading to bias in derived HR metrics. This limitation is especially problematic for running on hilly terrain, as frequent climbs and descents lead to changing effort [15, 16] and intricate HR curves.

Beyond purely longitudinal methods, generic multivariable deep learning approaches harness cross-variable information to reconstruct missing values [17, 18]. However, these approaches are not tailored to HR dynamics and require extensive training data, limiting practical use. Moreover, available implementations, such as the BRITS model, require training and testing time series to be of equal length [17, 19], a requirement that is seldom met in sports practice.

To overcome these limitations, this study presents a multivariable approach tailored to reconstruction of HR. It integrates energy-expenditure estimation, modeled HR dynamics, and drift correction. This HR model approach accounts for both longitudinal (temporal) and cross-variable dependencies. Specifically, Global Navigation Satellite System (GNSS) data, which is commonly recorded in outdoor running, is leveraged to reconstruct missing HR samples.

The aim of this study is to investigate the HR model approach for reconstructing missing HR data in trail running. Reconstruction accuracy is assessed for different exercise intensity profiles and missing-data lengths, providing a novel, systematic evaluation of model performance. It is hypothesized that the HR model approach performs better than current methods under non-constant exercise intensity, and that this improvement is larger for longer missing-data intervals.

## Methods

The accuracy of the HR reconstruction methods is assessed on experimentally collected data. Data collection and preprocessing are described in Data collection and Preprocessing sections, followed by the systematic procedure to artificially introduce missing data into the HR recordings (Missing data procedure section). HR model approach section presents the HR model approach for reconstructing missing data and Statistical analysis section details the statistical analysis. The data processing and reconstruction methods were implemented in Python 3.11 and the statistical processing was performed in R 4.4.3.

### Data collection

Data was collected from 12 recreational adult trail runners, recruited from members of the Trail Running Association of Queensland (male: n=8, age 40.8±8.8 years, height 178.9±5.5 cm, weight 74.8±7.4 kg; female: n=4, age 51.5±6.8 years, height 162.5±6.2 cm, weight 63.3±4.2 kg; characteristics given as mean ± SD). The participants performed a self-paced warm-up on a flat section of trail. Afterwards, the participants ran on a standardized outdoor track on Mt. Cootha (Brisbane, Australia). The track was a 2.1 km long with 165 m of elevation gain. Under the supervision of the researchers, participants were instructed to perform five bouts of this track, with the option to stop at any time. Between the bouts the participants rested until their HR was below 100 beats per minute (bpm).

In the first, fourth and fifth bout, the participants were able to freely select their gait pattern. In bout two the participants were asked to strictly run and in bout three they were instructed to strictly walk. This design was motivated by a second objective of this data collection: analyzing gait patterns across different inclinations. However, that analysis is beyond the scope of the present study, and walking and running are therefore treated interchangeably. This choice is supported by the fact that the researchers observed grounded running at higher inclines among many participants. Grounded running is a running-like movement, with no distinct flight phase, further blurring the boundary between walking and running [20].

In total 53 bouts were recorded. Eight participants performed all five bouts and due to exhaustion one subject performed four bouts and three subjects performed three bouts. The participants’ beat-by-beat HR was recorded using a 130 Hz ECG chest strap (H10, Polar Electro Oy, Kempele, Finland), which was worn according to the device’s user manual [21]. The H10 is especially suitable for measuring HR during physical activity [22]. Additionally, the runners position was captured with a 1 Hz GNSS receiver (GPSMAP 67i, Garmin Ltd, Olathe, USA) worn in the chest pocket of a running vest. Previous research suggests that GNSS receivers are suitable for dynamic running contexts [23]. Moreover, the GPSMAP 67i outperformed other consumer devices in outdoor terrain [24]. Data collection was approved by the Human Research Ethics Committee of The University of Queensland (reference number: 2025/HE000556) and conducted in accordance with the declaration of Helsinki. All participants gave their written informed consent prior to participation and data was recorded between April and November 2025.

### Preprocessing

The HR monitor provided data as beat-by-beat RR intervals in milliseconds. The RR intervals are a one-dimensional list, with no explicit timestamp. The timestamp was determined by the cumulative sum of the RR intervals. Therefore, missing samples manifest as unusually long intervals, instead of null values. Ectopic beats were removed using the Python package hrv-analysis [25]. Furthermore, HR was converted to bpm for better interpretability.

To ensure that reconstruction accuracy is only assessed on reliable HR data, the following quality check was performed for each bout. Based on previous studies, it was assumed that HR between two consecutive beats cannot change by more than 10 bpm [26, 27]. A bout was excluded if the threshold was exceeded more than ten times. Given the average bout duration of 21 minutes, the researchers deemed this limit a reasonable balance between data quality and inclusion. 12 bouts were excluded through this quality check. Note, that this rule may flag not only missing values but also other measurement artefacts (e.g., wrongly detected beats). A detailed characterization of measurement artefacts in HR recordings is provided in [9].

The GNSS receiver provided the participants latitude, longitude and altitude at 1 Hz. From this, the distance traveled in meters was calculated as the geodesic distance using the geopy Python package [28]. Because the running track had a considerable inclination, the three dimensional distance traveled was calculated. It was determined as the hypotenuse of the right-angled triangle whose legs are the distance traveled and altitude difference (in meter). Additionally, the participants’ velocity was determined from the distance traveled ($\frac{\Delta distance}{\Delta time}$). Lastly, GNSS data was upsampled to the higher-frequency HR timestamps via linear interpolation. All four bouts of participant 12 were excluded due to measurement errors that caused altitude readings to jump by more than 4 m within one second.

### Missing data procedure

To simulate missing data, intervals were systematically removed from the HR. These artificial HR gaps were reconstructed independently, with the rest of the data available as context. Reconstruction accuracy was assessed by comparing the reconstructed values to the ground-truth HR within the removed intervals.

**Fig. 1:**
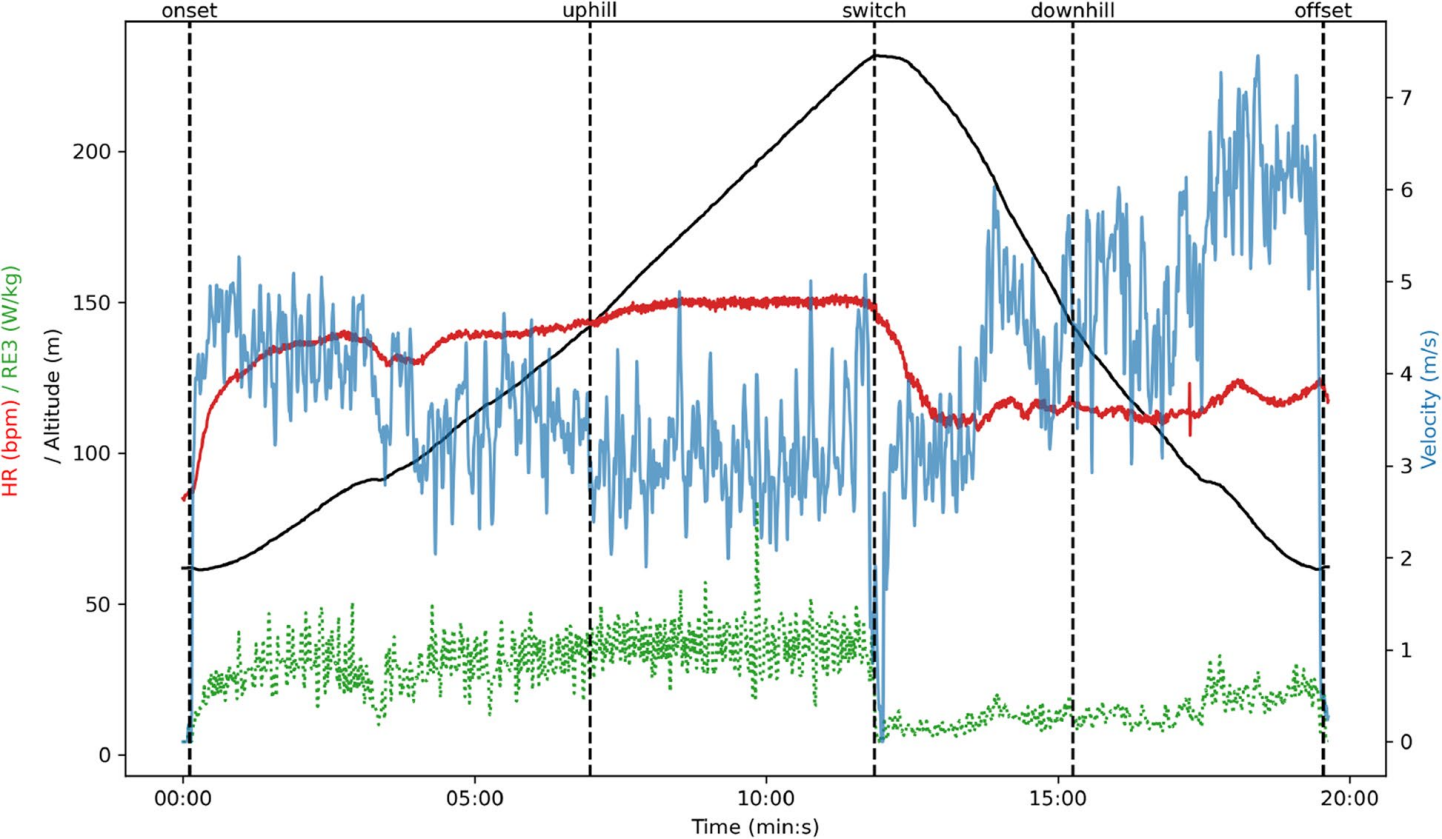
Recorded data from participant 10 bout 3. The red line is the HR, the black line is the altitude, the blue line is the velocity and the dotted green line is the estimated energy expenditure (RE3). The dashed lines indicate the points of the intensity profiles (from left to right): *onset*, *uphill*, *switch*, *downhill* and *offset*

In every bout, missing data was simulated at five phases, that each represent exercise intensity profiles common to trail running. The *onset* and *offset* were defined as the tenth and tenth-to-last sample of the bout. Taking the tenth samples guarantees sufficient HR values before and after the gaps to employ offline reconstruction methods. After the first 80 m of ascend, a phase of quasi-constant intensity *uphill* running was reached. This corresponds to about half of the total elevation gain and represents a phase in which the HR showed only little variation. Analogously, constant intensity *downhill* running was defined after the first 80 meters of descent. At the highest elevation, the inclination *switched* from uphill to downhill, causing a drop in exercise intensity. Fig. 1 shows HR, altitude and velocity for a randomly selected bout, highlighting the points used to define the intensity profiles: *onset*, *uphill*, *switch*, *downhill*, and *offset*.

At each of the five intensity phases, different lengths of consecutively missing data were considered. The missing-data lengths were 1, 10, 25, 50, 100, 200, 400 and 800 missing samples. 800 was the highest missing-data length, as longer gaps would lead to overlap between intensity phases. For *uphill*, *switch* and *downhill* the missing-data intervals were centered around the points defined above. For *onset* and *offset*, the intervals were set after and before the defined points, respectively.

It should be noted that against the intentions of the researchers, the *offset* intensity profile showed inconsistencies. Upon inspection of data it became evident that participants behaved differently at the end of the bouts. In some bouts participants performed a “final push”, likely encouraged by the downhill, while other times they finished the bouts at a recovery pace. To avoid distortions in the statistical estimates due to data inconsistencies, the *offset* intensity profile was excluded from the primary analysis. Appendix A: Table 4, Fig. 5, Tables 5 and 6 report the results including the *offset* intensity profile, demonstrating that this did not alter the main findings.

### HR model approach

The HR model approach for reconstructing missing HR values consists of three steps: energy-expenditure estimation, modeling HR dynamics, and drift correction. A key difference to current longitudinal methods is, that additional information, besides adjacent HR values, is leveraged for reconstruction. In outdoor running, GNSS data is commonly recorded alongside HR via smart watches or smartphones, making it a convenient resource for reconstruction.

#### Energy-expenditure estimation

For estimating metabolic energy-expenditure ($\dot{M}$ in $\textrm{W}\cdot \textrm{kg}^{-1}$), the recent running energy-expenditure estimation (RE3) was used [29]. This estimation equation depends on running velocity (*S*) and the inclination (*G*, as decimal):

1 $$\begin{aligned} \dot{M} = 4.43 + 1.51 \times S + 0.37 \times S^2 + 30.4269 \times S \times G (1 - 1.1334^{(1 - 1.0563^{(100 \times G + 43.4446)}})) \end{aligned}$$

The coefficients were determined by [29] based on aggregated data from multiple treadmill studies. Compared to similar estimation models [30–32], RE3 showed higher accuracy, and it is also applicable to *downhill* running [29].

In the present study, energy expenditure is estimated for each sample based on the GNSS-derived external load. Note, that the used velocity was based on the distance traveled in three dimensions, which is consistent with the velocity on an inclined treadmill. Fig. 1 shows RE3 for a randomly selected bout. Despite a higher velocity during the *downhill*, there was a notable drop in the energy expenditure when the inclination switched from uphill to downhill, which aligns with the notion that running downhill is easier. Similarly, HR showed lower values during *downhill* (Fig. 1). But unlike RE3, which reacts instantaneously to changes in velocity or inclination, HR exhibits a gradual response to changes [14]. Furthermore, HR dynamics differ between individuals.

#### HR model

An individualized approach was used, that models HR dynamics from energy expenditure. While different HR models have been proposed in literature [33–36], a differential equation approach was used, because it relies on few interpretable parameters and only requires a single initial value instead of an interval of preceding strain [37]. According to [37] HR was modeled as

2 $$\begin{aligned} \frac{dhr_t}{dt} = \frac{K}{\tau } \times p_t - \frac{hr_t - hr_{eq}}{\tau } \end{aligned}$$

$p_t$ denotes the excitation variable, which in the present study is RE3. $hr_t$ is the HR, and $\frac{dhr_t}{dt}$ is its time derivative. The $hr_{eq}$ parameter represents the equilibrium HR, which is the HR in the absence of workload. The gain parameter *K* indicates the proportionality between a given increase in power and the corresponding increase in HR. $\tau$ is the characteristic time of the HR model, describing the delay of the HR response.

The differential equation Eq. 2 was solved numerically using the SciPy implementation of the Runge-Kutta-Fehlberg solver RK45 [38, 39] to get HR for each timestep. This solver integrates the equation given an initial HR value ($hr_{init}$), which was set to the last known HR value before the missing data.

The model parameters $hr_{eq}$, *K*, $\tau$ were fitted for each participant individually in a leave-one-bout-out procedure. Iteratively, model parameters were fitted for all but one bout of a participant. The determined parameters were then used to reconstruct HR in the bout that was not used during fitting.

The objective of fitting was to find parameters that minimize the root mean square error (RMSE) between the modeled HR and measured HR. For this, the SciPy implementation of differential evolution was used, because it is robust, versatile and allows for parallelization [39, 40]. The maximum number of iterations and the population size are the control variables for differential evolution and were set to 200 and 20, respectively. Furthermore, the tolerance was set to 0 to avoid early termination, and the random seed was set to 42 for repeatability.

To ensure the fitting procedure results in stable parameters, it was tested through repeated fittings with random initial condition. The HR model was fitted 20 times for bout two to five of participant 1. This is the same data segmentation used for reconstructing HR in bout one. Overall, the parameters and fitting RMSE showed low variation, indicating that the fitting procedure is appropriate (mean ± SD: $hr_{eq} = 112.37 \pm 1.1$ bpm, $K = 1.16 \pm 0.04$ $\textrm{bpm}\cdot \textrm{kg}\cdot \textrm{W}^{-1}$, $\tau = 39.58 \pm 3.96$ s, and RMSE $5.71 \pm 0.05$).

In the following the HR model approach is referred to as HRM.

#### Drift correction

**Fig. 2:**
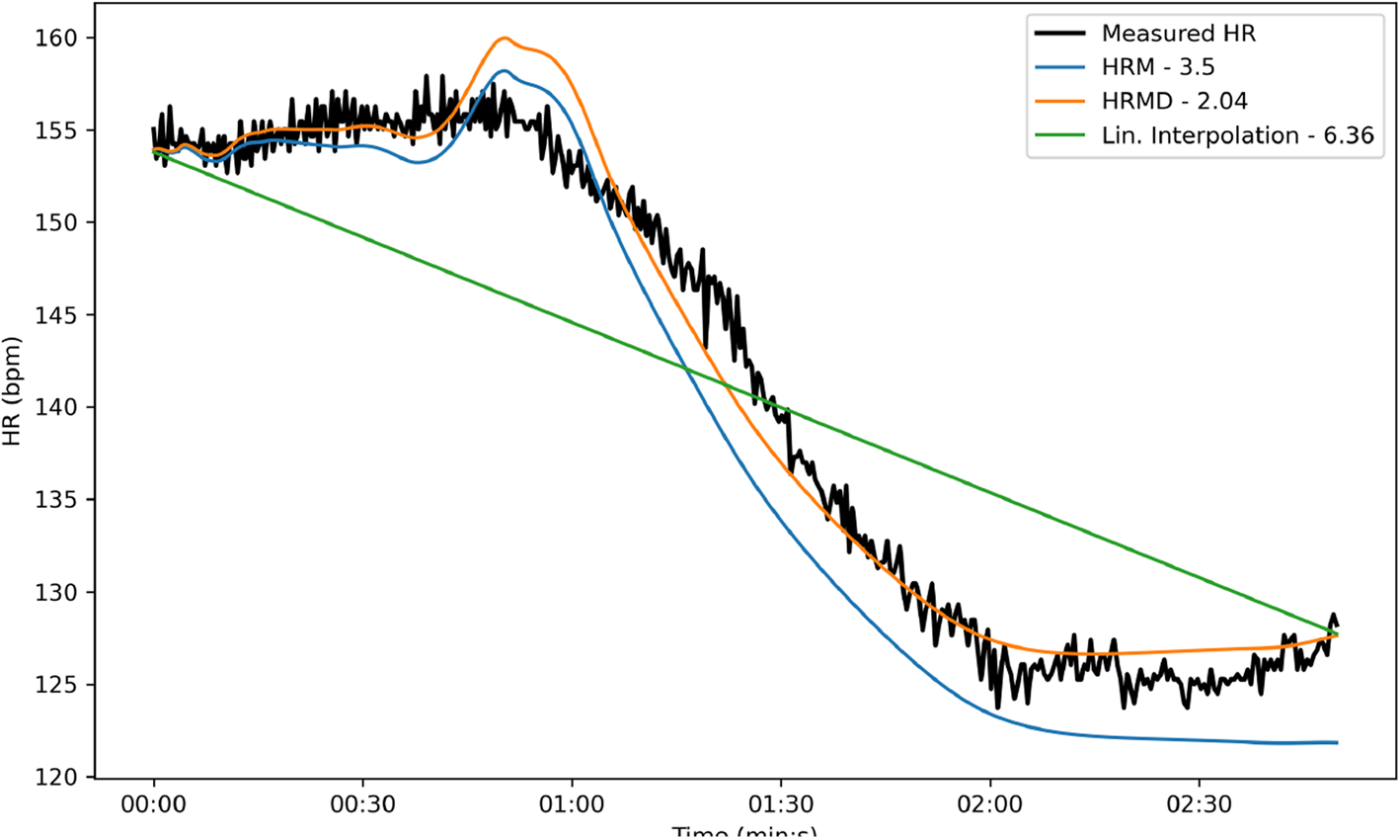
Example missing data interval of 400 samples at *switch* from uphill to downhill. Besides the actual HR (black line) the reconstructions from HRM (blue), HRMD (orange), and linear interpolation (green) are shown. The RMSE between the reconstructed and actual HR is given in the figure legend

Figure 2 shows an example of the HR reconstruction using HRM. While the HR model follows the overall shape of the actual HR, the difference increases with time. To counter this, a drift correction was applied.

To quantify drift, the difference between the next known HR value after the gap and the last reconstructed value was determined: $\Delta = \textrm{hr}_{\text {post}} - \textrm{hr}_{\text {last}}$. Assuming the drift is linear, a correction was applied to each reconstructed HR value ($\textrm{hr}_{\text {rec}}$). For the *i*-th sample in a gap of length *N* (with $i=0,1,...,N$) the corrected HR is

3 $$\begin{aligned} \textrm{hr}_{\text {corr}}(i) = \textrm{hr}_{\text {rec}}(i) + \frac{i}{N}\times \Delta . \end{aligned}$$

This leaves the beginning of the gap unchanged ($i=0$) and aligns the end of the gap with the post-gap measurement ($i=N$). In Fig. 2, the reconstructed HR with drift correction is very similar to the actual HR.

In the following the HR model with drift correction is referred to as HRMD.

### Statistical analysis

Reconstruction accuracy of the proposed HRM and HRMD was compared with current longitudinal imputation methods. For each reconstructed gap, error magnitude was quantified by the RMSE between the reconstructed and actual heart rate. A linear mixed-effects model was used to compare RMSE across reconstruction methods, missing-data lengths and intensity profiles. To account for variability between participants, a random intercept was added. For the residuals to be normally distributed, RMSE was log-transformed, with an offset of 1 to avoid numerical errors if *log*(0).

The model was fitted with the lmerTest package in R. Pairwise contrasts were computed using the emmeans package with Tukey adjustment for multiple comparisons, Cohen’s d effect size, and Satterthwaite approximation for degrees of freedom. Results were deemed statistically significant if $p<0.05$. Diagnostics indicate that the model was appropriate. The model residuals showed no systematic trend over fitted values, and the residuals, as well as random intercepts were approximately normal distributed (see Appendix B: Figs. 6, 7, 8 and 9).

Due to the novelty of the research and because previous studies did not provide sufficient statistical details, an a priori power analysis was not possible. Instead, to assess the adequacy of the design (number of participants and repeated-measures per participant), the minimum detectable effect size (MDES) was examined for pairwise contrasts from the linear mixed-effects model at 80% power. Following [41], standardized MDES was reported as

4 $$\begin{aligned} \textrm{MDES} = \frac{(c_{\alpha } + t_{0.8,df})\textrm{SE}}{\sigma _e} \end{aligned}$$

where $c_{\alpha }$ is the Tukey critical component (which is equivalent to the Tukey-adjusted margin of error divided by $\textrm{SE}$, $\alpha =0.05$), $t_{0.8,df}$ is the 0.8 quantile of the central t distribution with *df* degrees of freedom, $\textrm{SE}$ is the standard error of the contrast, and $\sigma _e$ is the residual standard deviation.

In addition to linear mixed-effects model analysis, the mean error (ME) was used to assess bias across all samples. The 90% limits of agreement (LoA, defined as 1.645 times the standard deviation) were also reported to indicate the range within which 90% of the differences are expected to fall.

The presented results focus on the comparison of HRM/HRMD and linear interpolation. Linear interpolation showed good results in previous comparison studies [9–12], and it also outperforms other longitudinal imputation methods on the present data (see Appendix C: Fig. 10, Tables 7 and 8). In the following, linear interpolation is referred to as LI.

The intra-class correlation coefficient (ICC) and coefficient of variation (CV) for the RMSE across repeated bouts are reported in Appendix D, Table 9. However, given the low RMSE values, these metrics have to be interpreted with caution. ICC, which reflects the ratio of between-cluster to total variance, can appear low because between-cluster variance is bounded at zero, despite small absolute errors [42, 43]. CV, calculated as the ratio of the standard deviation to the mean, can become large when the mean is low [44]. Thus, in this case low ICC/CV values do not necessarily imply poor practical repeatability.

## Results

**Fig. 3:**
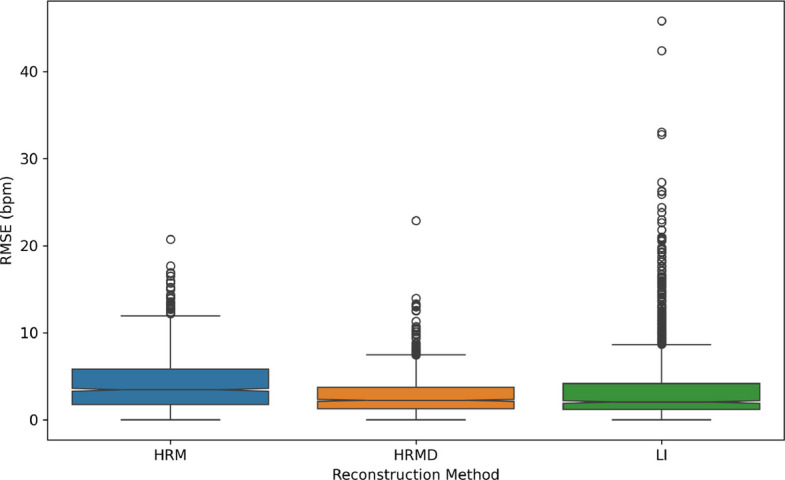
Boxplot of RMSE, aggregated over all missing-data lengths and intensity profiles. The notches represent the 95% confidence interval around the median

Figure 3 shows the RMSE of HRM, HRMD and LI, aggregated over all missing-data lengths and intensity profiles. HRMD and LI showed a similar median RMSE of around 2 bpm. The median RMSE of HRM was around 3 bpm. The pairwise contrasts are listed in Table 1. They revealed that HRMD and LI had a significantly lower RMSE than HRM. Furthermore, HRMD had a significantly lower RMSE than LI.

**Table 1:** Pairwise contrasts between the reconstruction methods, aggregated over all missing-data lengths and intensity profiles

Contrast	Estimate	CL	t	p	d
HRM - HRMD	0.19	[0.15, 0.22]	12.58	<0.0001	0.52
HRM - LI	0.10	[0.07, 0.14]	6.97	<0.0001	0.29
HRMD - LI	-0.08	[-0.12, -0.05]	-5.61	<0.0001	-0.23

For all rows, the degrees of freedom are 3446.853

**Fig. 4:**
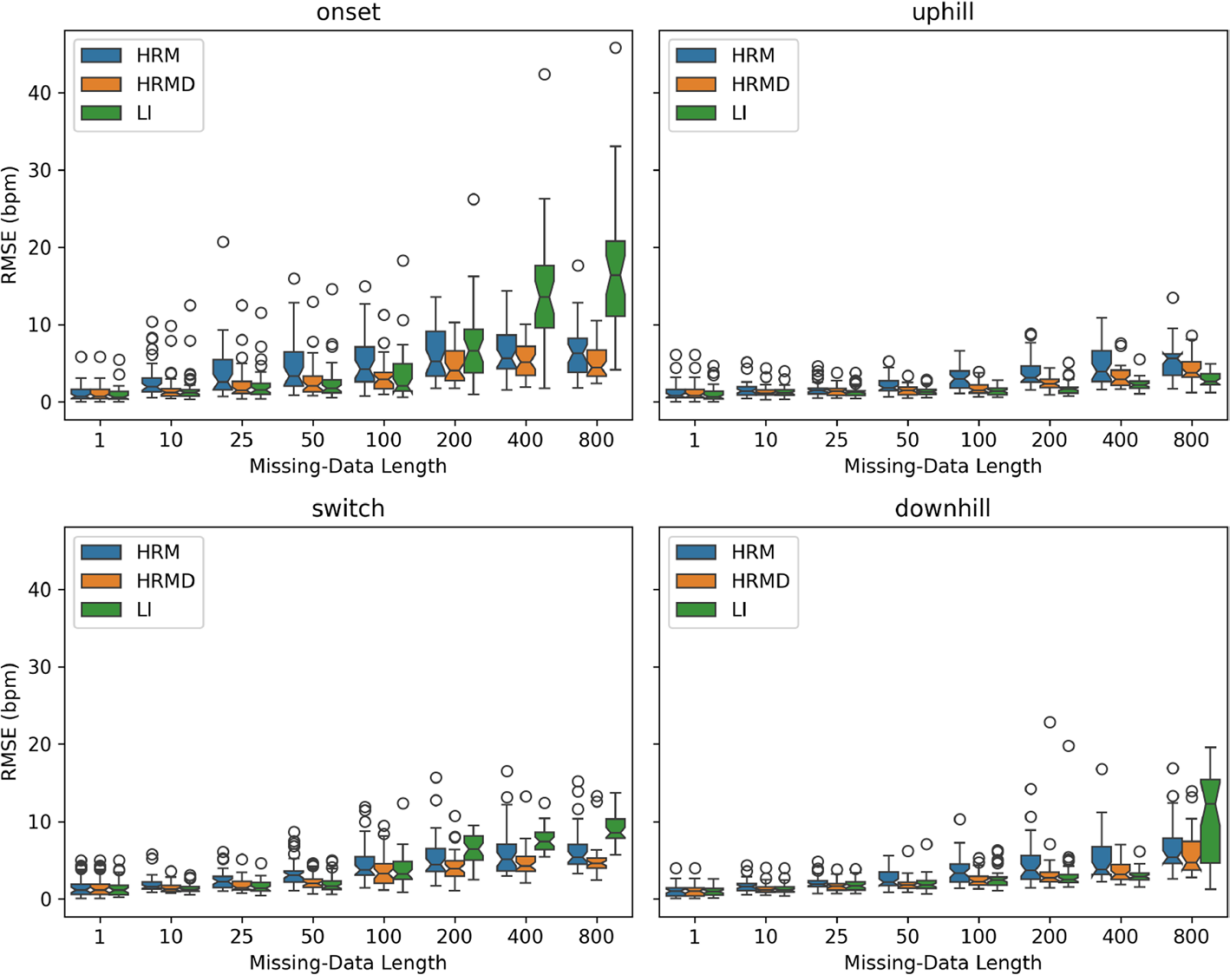
Boxplot of the RMSE ordered by missing-data lengths and intensity profiles. The notches represent the 95% confidence interval around the median

**Fig. 5:**
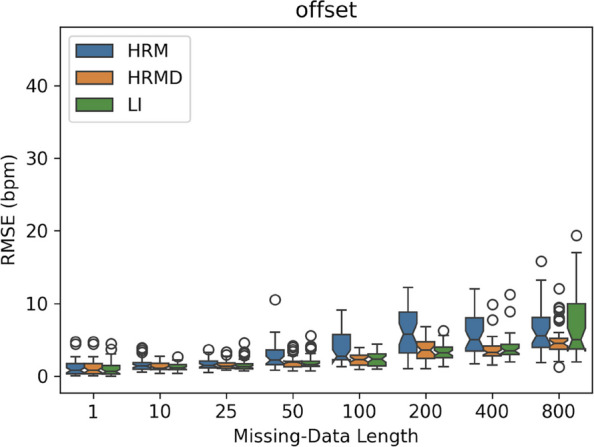
Boxplot of the RMSE for the *offset* intensity profile ordered by missing-data lengths. The notches represent the 95% confidence interval around the median

**Fig. 6:**
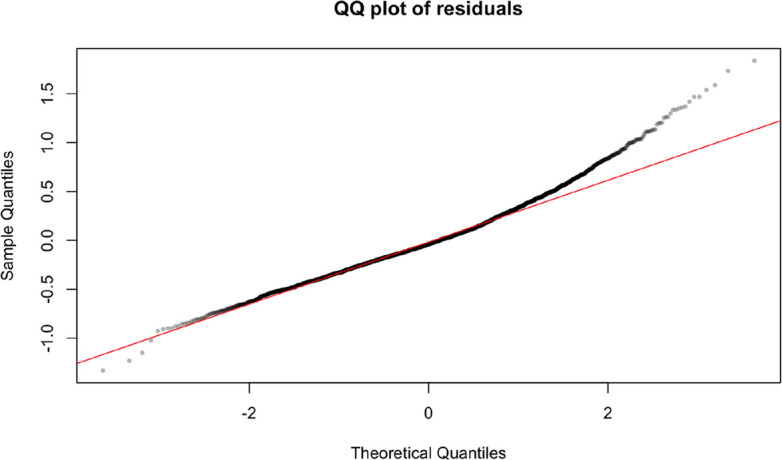
QQ plot of the model residuals. The line of equality is indicated in red

**Fig. 7:**
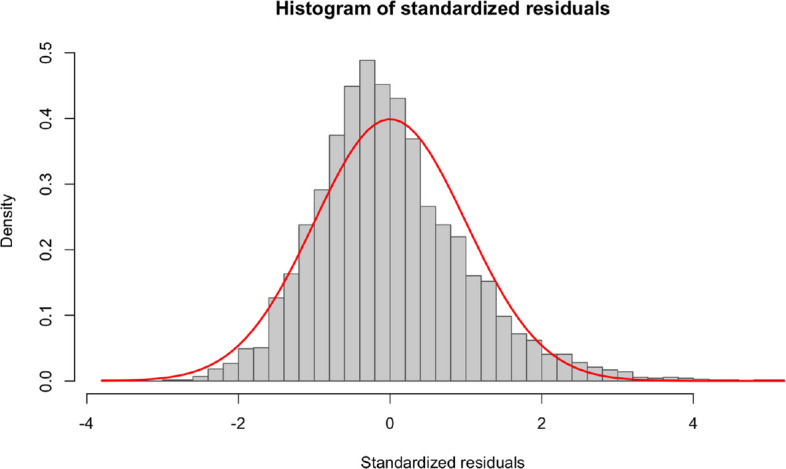
Histogram of the standardized model residuals. The red line indicates the standard normal distribution

**Fig. 8:**
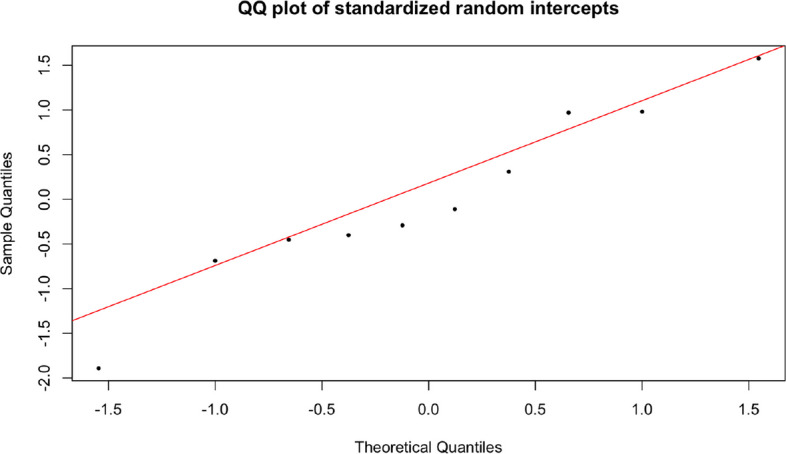
QQ plot of the random intercepts. The line of equality is indicated in red

**Fig. 9:**
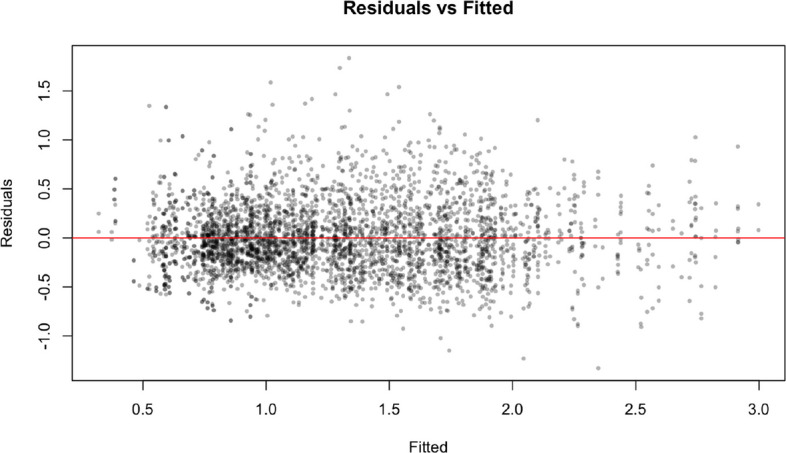
Scatter plot of residuals over the fitted values. The red line is the horizontal reference

**Fig. 10:**
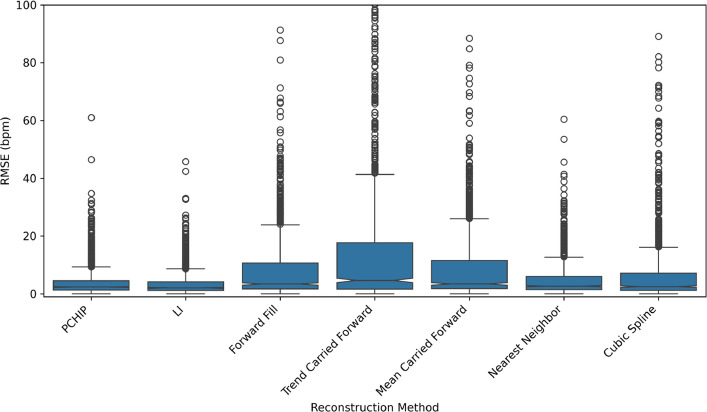
Boxplot of the RMSE of all longitudinal imputation methods, aggregated over all missing-data lengths and intensity profiles. Extreme outliers of Cubic Spline and Trend Carried Forward are cutoff for better visibility. The notches represent the 95% confidence interval around the median

However, the results in Fig. 3 may hide an underlying structure, since all fixed effects of the model and their interactions were significant (see Appendix Table 10 for test statistics). Therefore, Fig. 4 shows the the RMSE of the reconstruction methods ordered by missing-data lengths and intensity profiles. RMSE distribution showed noticeable differences depending on the condition. The pairwise contrasts (Table 2) revealed that HRMD had a significantly lower RMSE than LI for the *onset* and *switch* profiles when $\ge$200 samples were missing. The effect increased for higher missing-data lengths. HRMD was also significantly better than LI for the *downhill* profile at 800 missing samples. LI outperformed HRMD for the *uphill* profile. However, the effect size was smaller compared to the other cases. Furthermore, HRMD was significantly better than HRM in many conditions, and there was no condition in which HRM was superior over HRMD. The comparison between HRM and LI was less clear. LI was significantly better than HRM in many conditions. HRM was only significantly better than LI for the *downhill*, *switch*, and *onset* profiles with missing-data lengths of 800, $\ge$200, and $\ge$400, respectively. Each contrast of the linear mixed-effects model has $df=3446.853$ degrees of freedom and a standard error of $\textrm{SE}=0.084$. Therefore, $\textrm{MDES}=0.74$ for all contrasts.

**Table 2:** All significant pairwise contrasts between the reconstruction methods, ordered by missing-data lengths and intensity profiles

Contrast	Intensity profile	Missing-data length	Estimate	CL	t	p	d
HRMD - LI	*downhill*	800	-0.48	[-0.68, -0.28]	-5.71	<0.0001	-1.33
HRMD - LI	*switch*	200	-0.39	[-0.59, -0.19]	-4.64	<0.0001	-1.08
HRMD - LI	*switch*	400	-0.47	[-0.67, -0.28]	-5.64	<0.0001	-1.31
HRMD - LI	*switch*	800	-0.55	[-0.74, -0.35]	-6.52	<0.0001	-1.51
HRMD - LI	*onset*	200	-0.31	[-0.51, -0.12]	-3.75	0.0005	-0.87
HRMD - LI	*onset*	400	-0.81	[-1.01, -0.62]	-9.67	<0.0001	-2.25
HRMD - LI	*onset*	800	-1.02	[-1.22, -0.82]	-12.12	<0.0001	-2.82
HRMD - LI	*uphill*	200	0.22	[0.03, 0.42]	2.67	0.0207	0.62
HRMD - LI	*uphill*	400	0.25	[0.05, 0.45]	2.99	0.0080	0.69
HRMD - LI	*uphill*	800	0.27	[0.07, 0.46]	3.17	0.0044	0.74
HRM - HRMD	*downhill*	100	0.24	[0.04, 0.44]	2.85	0.0123	0.66
HRM - HRMD	*downhill*	200	0.23	[0.03, 0.42]	2.68	0.0201	0.62
HRM - HRMD	*downhill*	400	0.25	[0.06, 0.45]	3.03	0.0070	0.70
HRM - HRMD	*switch*	25	0.23	[0.03, 0.42]	2.70	0.0189	0.63
HRM - HRMD	*switch*	50	0.26	[0.06, 0.46]	3.12	0.0052	0.72
HRM - HRMD	*onset*	10	0.28	[0.08, 0.48]	3.34	0.0025	0.78
HRM - HRMD	*onset*	25	0.35	[0.16, 0.55]	4.20	<0.0001	0.98
HRM - HRMD	*onset*	50	0.37	[0.17, 0.56]	4.35	<0.0001	1.01
HRM - HRMD	*onset*	100	0.41	[0.21, 0.61]	4.87	<0.0001	1.13
HRM - HRMD	*onset*	200	0.21	[0.01, 0.4]	2.45	0.0384	0.57
HRM - HRMD	*uphill*	100	0.35	[0.15, 0.55]	4.17	<0.0001	0.97
HRM - HRMD	*uphill*	200	0.29	[0.1, 0.49]	3.49	0.0014	0.81
HRM - HRMD	*uphill*	400	0.25	[0.05, 0.44]	2.95	0.0091	0.69
HRM - LI	*downhill*	100	0.21	[0.01, 0.4]	2.47	0.0366	0.57
HRM - LI	*downhill*	200	0.26	[0.07, 0.46]	3.12	0.0051	0.73
HRM - LI	*downhill*	400	0.39	[0.19, 0.58]	4.60	<0.0001	1.07
HRM - LI	*downhill*	800	-0.37	[-0.57, -0.17]	-4.41	<0.0001	-1.02
HRM - LI	*switch*	25	0.27	[0.07, 0.46]	3.17	0.0044	0.74
HRM - LI	*switch*	50	0.32	[0.12, 0.51]	3.78	0.0005	0.88
HRM - LI	*switch*	200	-0.20	[-0.4, 0]	-2.40	0.0439	-0.56
HRM - LI	*switch*	400	-0.28	[-0.48, -0.08]	-3.33	0.0025	-0.77
HRM - LI	*switch*	800	-0.37	[-0.57, -0.18]	-4.46	<0.0001	-1.04
HRM - LI	*onset*	10	0.29	[0.09, 0.49]	3.43	0.0018	0.80
HRM - LI	*onset*	25	0.38	[0.18, 0.58]	4.53	<0.0001	1.05
HRM - LI	*onset*	50	0.42	[0.23, 0.62]	5.04	<0.0001	1.17
HRM - LI	*onset*	100	0.40	[0.2, 0.6]	4.75	<0.0001	1.10
HRM - LI	*onset*	400	-0.68	[-0.88, -0.48]	-8.08	<0.0001	-1.88
HRM - LI	*onset*	800	-0.86	[-1.05, -0.66]	-10.18	<0.0001	-2.37
HRM - LI	*uphill*	50	0.26	[0.06, 0.45]	3.06	0.0063	0.71
HRM - LI	*uphill*	100	0.45	[0.26, 0.65]	5.38	<0.0001	1.25
HRM - LI	*uphill*	200	0.52	[0.32, 0.71]	6.16	<0.0001	1.43
HRM - LI	*uphill*	400	0.50	[0.3, 0.7]	5.93	<0.0001	1.38
HRM - LI	*uphill*	800	0.45	[0.26, 0.65]	5.38	<0.0001	1.25

For all rows, the degrees of freedom are 3446.853

Table 3 shows the ME and the LoA for the reconstruction methods in each condition. The corresponding Bland Altman plots can be found in the Appendix F (Figs. 11, 12, 13, 14, 15, 16, 17, 18, 19, 20, 21, 22, 23, 24 and 25). For HRM, highest ME deviation from zero was 2.29 bpm for *onset* with 200 missing samples. The LoA tended to increase with longer missing-data intervals, reaching a maximum of 12.06 bpm at *onset* with 400 missing samples. HRMD showed ME values close to zero, with a maximum deviation of 2.25 bpm. Throughout all conditions, the LoA of HRMD stayed under 10 bpm.

**Table 3:** Mean error (± SD) between actual and reconstructed HR over all samples

Intensity profile	Missing-data length	HRM	HRMD	LI
*downhill*	1	0.42 ± 1.29 (2.12)	0.42 ± 1.29 (2.12)	0.56 ± 1.06 (1.75)
*downhill*	10	-0.12 ± 1.88 (3.10)	0.10 ± 1.51 (2.49)	0.16 ± 1.51 (2.48)
*downhill*	25	0.06 ± 2.17 (3.56)	0.70 ± 1.68 (2.77)	0.87 ± 1.72 (2.84)
*downhill*	50	-0.32 ± 2.89 (4.76)	0.24 ± 2.12 (3.49)	0.42 ± 2.24 (3.68)
*downhill*	100	-0.27 ± 4.01 (6.60)	0.35 ± 2.62 (4.30)	0.46 ± 2.88 (4.73)
*downhill*	200	-0.25 ± 5.33 (8.76)	0.74 ± 4.81 (7.91)	0.57 ± 4.32 (7.11)
*downhill*	400	-0.55 ± 6.29 (10.35)	0.22 ± 3.87 (6.37)	-0.16 ± 3.10 (5.10)
*downhill*	800	-0.84 ± 7.30 (12.01)	2.01 ± 5.92 (9.74)	-8.02 ± 9.18 (15.11)
*onset*	1	0.19 ± 1.58 (2.60)	0.19 ± 1.58 (2.60)	0.11 ± 1.45 (2.39)
*onset*	10	0.58 ± 3.59 (5.91)	-0.12 ± 2.60 (4.27)	-0.30 ± 2.87 (4.71)
*onset*	25	1.33 ± 5.13 (8.44)	0.44 ± 3.24 (5.33)	0.35 ± 3.03 (4.98)
*onset*	50	1.75 ± 5.53 (9.09)	0.41 ± 3.62 (5.95)	0.66 ± 3.64 (5.99)
*onset*	100	2.29 ± 6.41 (10.55)	1.00 ± 3.81 (6.26)	2.28 ± 4.45 (7.31)
*onset*	200	1.43 ± 6.92 (11.38)	2.25 ± 4.78 (7.86)	6.26 ± 6.03 (9.93)
*onset*	400	-1.16 ± 7.33 (12.06)	0.48 ± 5.81 (9.56)	12.63 ± 9.89 (16.26)
*onset*	800	-1.45 ± 7.2 (11.84)	-0.60 ± 5.52 (9.08)	14.56 ± 12.31 (20.25)
*switch*	1	0.49 ± 2.04 (3.36)	0.49 ± 2.04 (3.36)	0.55 ± 1.80 (2.96)
*switch*	10	0.13 ± 2.13 (3.51)	-0.29 ± 1.57 (2.58)	-0.21 ± 1.51 (2.49)
*switch*	25	0.52 ± 2.71 (4.46)	-0.41 ± 1.89 (3.11)	-0.02 ± 1.83 (3.01)
*switch*	50	1.39 ± 3.54 (5.82)	-0.11 ± 2.47 (4.06)	0.76 ± 2.24 (3.69)
*switch*	100	0.37 ± 5.39 (8.86)	-0.06 ± 4.12 (6.78)	2.33 ± 3.60 (5.93)
*switch*	200	0.22 ± 6.07 (9.98)	0.88 ± 4.49 (7.39)	3.91 ± 5.45 (8.96)
*switch*	400	0.08 ± 6.78 (11.15)	0.46 ± 4.96 (8.16)	3.15 ± 7.24 (11.9)
*switch*	800	0.30 ± 6.81 (11.21)	0.41 ± 5.39 (8.86)	1.83 ± 9.07 (14.93)
*uphill*	1	-0.38 ± 1.77 (2.91)	-0.38 ± 1.77 (2.91)	-0.20 ± 1.52 (2.51)
*uphill*	10	-0.08 ± 1.86 (3.07)	-0.08 ± 1.54 (2.54)	-0.16 ± 1.48 (2.44)
*uphill*	25	-0.18 ± 1.86 (3.06)	0.00 ± 1.65 (2.71)	-0.07 ± 1.52 (2.50)
*uphill*	50	-0.07 ± 2.40 (3.95)	0.11 ± 1.66 (2.73)	-0.10 ± 1.48 (2.44)
*uphill*	100	0.22 ± 3.32 (5.46)	0.38 ± 1.93 (3.17)	-0.20 ± 1.53 (2.51)
*uphill*	200	-0.26 ± 4.23 (6.96)	-0.52 ± 2.46 (4.05)	-0.40 ± 1.93 (3.18)
*uphill*	400	-0.50 ± 5.23 (8.60)	-0.88 ± 3.41 (5.62)	0.14 ± 2.41 (3.96)
*uphill*	800	-0.11 ± 5.90 (9.70)	-0.58 ± 4.49 (7.38)	1.00 ± 2.86 (4.71)

Furthermore, the 90% LoA are stated in brackets

LI exhibited high ME values at *onset*, with a maximum of 14.56 bpm for 800 missing samples. This condition also yielded the highest LoA, at 20.25 bpm. Notably, in the intensity profile *switch* with $\ge$200 missing samples, the LOA were relatively high, while ME remained below 4 bpm. In other intensity profiles and at lower missing-data lengths, LI showed ME values around zero and LoA values comparable to HRM and HRMD.

## Discussion

This study evaluated a HR reconstruction approach that integrates GNSS-derived load with a model of HR dynamics and a simple drift correction. The approaches were compared against LI, a commonly used longitudinal imputation method, across a range of gap lengths and exercise intensity profiles representative of trail running. Three main findings emerged: (1) overall, HRMD and LI achieved similarly low errors, with HRMD slightly outperforming LI; (2) the superiority of HRMD was condition-dependent: for long gaps during non-steady exercise intensity (*onset* and *switch*), HRMD showed lower RMSE, bias and dispersion; (3) LI remained slightly superior during steady *uphill* segments.

The results align with the central hypothesis, that the HR model approach provides an advantage over longitudinal imputation when intensity is non-constant and gaps are longer. Conversely, when intensity is relatively steady or gaps are short, simpler longitudinal methods, such as LI, remain strong baselines.

HRMD was significantly better than LI at *onset* and *switch* for gaps $\ge$ 200 samples, and also at the longest gaps in *downhill*. This aligns with physiological expectations. At a step change in exercise intensity, HR typically follows a first-order response towards a new steady state [14, 45]. HRMD appears to reflect these time-dependent HR kinetics well. By contrast, the straight line imposed by LI cannot reproduce them, yielding higher ME and wider LoA, particularly for long gaps.

During steady *uphill*, LI outperformed HRMD, though the effect was smaller than in the other pairwise contrasts between the two approaches. Under quasi-constant exercise intensity, pre- and post-gap HR values are similar and variation is low, which favors LI’s linearity. The inferior performance of HRMD may be due to GNSS measurement noise, which introduced artificial variation of the modeled HR, leading to slightly higher errors.

The HR model approach without drift correction (HRM) accumulated errors throughout the gaps. The drift correction of HRMD anchored the reconstructed data to the post-gap measurement, significantly reducing RMSE in many conditions. Furthermore, HRM was never better than HRMD, indicating that the drift correction effectively manages the observed drift. The advantage of HRM over HRMD and LI is that it does not require post-gap data, making it suitable for real-time reconstruction.

Previous studies recommend a target accuracy of 10 bpm for HR prediction [33, 46, 47]. The studies base this threshold on the typical width of HR zones (approx. 10% of the maximum HR, or 15 to 20 bpm) and argue that half that margin is sufficient for detailed training planning. Across conditions, HRMD displayed ME values close to zero and LoA grew moderately with missing-data length, but remained below 10 bpm. Thus, more than 90% of the reconstructed values are expected to fall within ± 10 bpm of the ME, satisfying the target accuracy. LI and HRM exceed the accuracy target at long gaps with non-constant exercise intensity.

Prior studies comparing longitudinal imputation methods for HR data reported good performance for LI in short gaps [9–12]. The results of the present study confirm this strength of LI, while highlighting its limitations for long intervals with non-steady intensity - scenarios underrepresented in previous studies but common to outdoor running. The present study showed that HR reconstruction in these scenarios can be improved by leveraging GNSS data and a model HR of dynamics [37]. The proposed approach aligns with previous works that couple internal and external load [33–36, 48–50], but differs in the explicit integration of an inclination sensitive energy-expenditure estimation (RE3) [29]. To the authors’ knowledge, this is the first systematic evaluation of a cross-variable HR reconstruction method that assesses accuracy across a range of missing-data lengths and intensity profiles.

### Minimum detectable effect size

Across most pairwise contrasts, MDES is smaller than the corresponding effect size d. This is especially true for the contrasts where HRMD outperformed LI, for which d substantially exceeds the MDES. Because MDES represents the smallest effect size detectable with 80% power, this pattern indicates that the study design (number of participants and repeated-measures per participant) is capable to detect relevant effects. The repeated-measures design improves estimation precision by reducing the standard error of the estimates ($\textrm{SE}$). While this is a strength for internal validity, it also means the inferences are most directly applicable to the specific population: adult trail runners. It is uncertain how the results generalize to different demographics and other running disciplines.

That said, caution is warranted in interpretation: MDES and d were derived from the same mixed-effects model, and observed effects can be upwardly biased in finite samples (the “winner’s curse”), especially when power is limited [51–53]. Consequently, the apparent margin by which d exceeds MDES may overstate the true difference, and results should be contextualized with this potential inflation in mind.

### Strengths and limitations

Data was collected under ecologically valid conditions from recreational trail runners with realistic GNSS and HR signals. Furthermore, the range of missing-data lengths and intensity profiles provide a nuanced picture of the strengths and weaknesses of the reconstruction methods. A limitation of the missing data procedure is that gaps were simulated at predefined phases. In practice, data loss is more chaotic and may coincide with periods of excessive sweating or sensor displacement caused by torso movements or ground impacts.

A strength of HRM and HRMD is the modular integration of energy-expenditure estimation, HR modeling, and drift correction. Within this framework, HR dynamics were modeled through a small set of physiologically meaningful parameters, making the model interpretable.

The energy-expenditure estimation RE3 has the strength of being inclination-sensitive, which is important in trail running where inclination influences energetic demands [29, 54]. This also makes RE3 transferable to a wide range of running modalities, including accelerated running [55–57]. Moreover, in other sports, such as rowing or cycling, the energy-expenditure estimation could be replaced by directly measured power. This modularity enables a wide range of applications for the HR model approach, which should be investigated in further studies.

A limitation of RE3 is that it was developed for treadmill data but was applied to GNSS data. Field conditions introduce GNSS noise, leading to altitude or distance errors which propagate into the reconstructed HR. This could also be a contributing factor to the observed drift in the HRM reconstructions. Noise may be reduced by employing higher frequency GNSS devices, additional inertial sensors, barometric altitude, or map-derived elevation. Another constraint is that energetics of all gait patterns were treated as equal. RE3 was specifically designed for running [29], but energetics differ between walking and running [58, 59]. Thus, applying RE3 to walking could introduce slight variations in the estimated energy-expenditure. In the future, HR reconstruction could be improved through gait-specific energy-expenditure estimation and a better understanding of energetics in grounded running [20].

As indicated by the MDES analysis (Minimum detectable effect size section), future research should evaluate the HR reconstruction approach in specific demographic groups and larger cohorts. In this context, the inconsistencies during the *offset* intensity profile (Missing data procedure section), highlight the need for careful consideration of the experimental protocol. In sports like cycling or rowing, the supervision of the protocol may be facilitated by access to directly measured power.

Another limitation is that the present study only considered one gap at a time. To better reflect real-world conditions, further studies should investigate reconstruction accuracy over multiple gaps, as well as the effect on downstream HR metrics in different scenarios.

Furthermore, studies should investigate the characteristics of the drift that HRM exhibited. Alternative drift correction strategies, such as Bayesian filtering or adaptive HR model parameters may improve real-time reconstruction. Similar strategies could be adapted to model varying environmental conditions, such as changing temperature and humidity, that influence the HR response [50, 60] but are not accounted for in the current HR model approach.

### Practical implications

Reconstruction of HR enables the analysis of data that would otherwise be unavailable. This can improve downstream HR metrics, such as training intensity distribution, HR recovery, or HR kinetics. For practical application it is advisable to adapt the reconstruction method to the conditions. This is in line with recommendations from a previous study [11]. LI remains a competitive reconstruction method for short gaps and steady exercise intensity. It is simple, computationally light and requires no fitting. For longer gaps and changing exercise intensity, for example at the onset of exercise or terrain transitions, HRMD reduces reconstruction error and bias.

HRMD requires GNSS data to determine speed and inclination, as well as participant specific model fitting using at least one bout. In practice HR monitors are commonly paired with smart watches or smartphones, conveniently providing synchronized external load data of every workout. Yet, the participant specific model fitting restricts the practical application to athletes who routinely record their workouts. If available, data from cardiopulmonary exercise testing and other standardized exercise protocols, which many athletes undergo regularly, could be used for fitting. That said, HRMD cannot reconstruct HR for new or unknown athletes.

In this study, 16 out of 53 bouts were excluded due to poor data quality. Similar data attrition may be expected in real-world workout recordings. Yet, for athletes who train frequently, sufficient data for fitting HRMD can be collected over time. Furthermore, the reconstruction methods can, in principle, be applied to data from optical HR monitors, which are more susceptible to errors and may exhibit higher rates of missing data than the ECG device used in the present study [2, 61].

## Conclusion

Linear interpolation remains a strong baseline for reconstructing short, steady gaps, but its errors grow and become biased for long gaps during non-steady exercise intensity. The proposed HRMD approach, which integrates GNSS-derived energy-expenditure estimation with individualized HR dynamics and a simple drift correction, substantially improves reconstruction in those challenging scenarios while maintaining low bias. These findings argue for a hybrid approach that chooses the reconstruction method based on the scenario. Further improvements in workload estimation and online parameter adaptation may extend these benefits to real-time applications.

## Figures and Tables

**Fig. 11:**
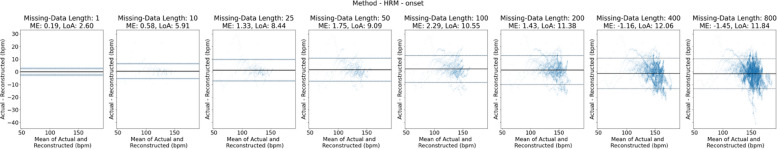
HRM - *onset*

**Fig. 12:**
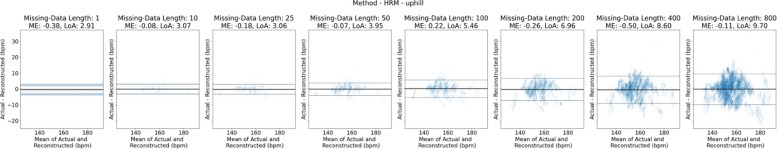
HRM *uphill*

**Fig. 13:**
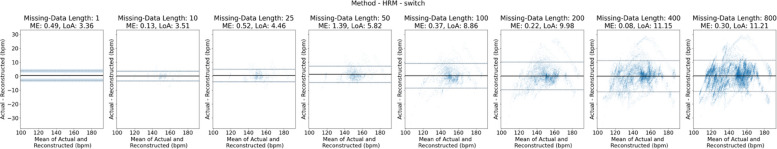
HRM - *switch*

**Fig. 14:**
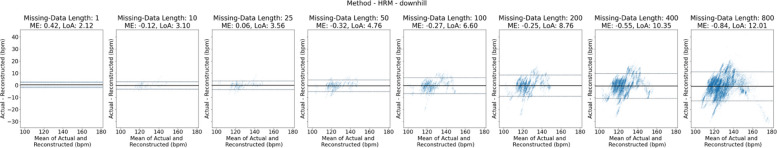
HRM - *downhill*

**Fig. 15:**
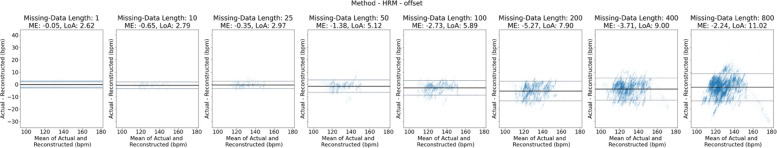
HRM - *offset*

**Fig. 16:**
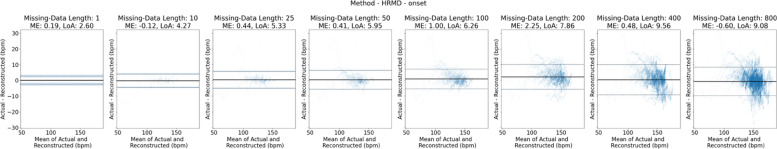
HRMD - *onset*

**Fig. 17:**
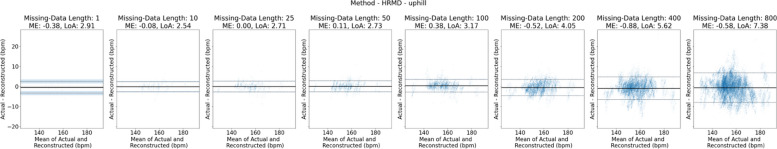
HRMD - *uphill*

**Fig. 18:**
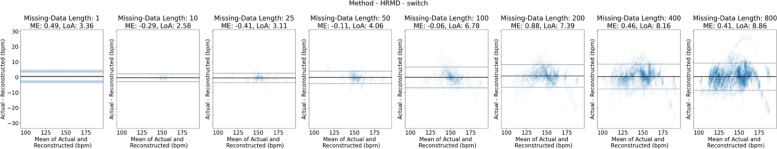
HRMD - *switch*

**Fig. 19:**
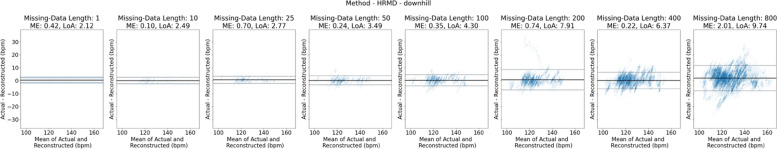
HRMD - *downhill*

**Fig. 20:**
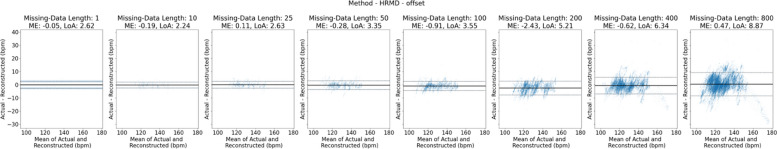
HRMD - *offset*

**Fig. 21:**
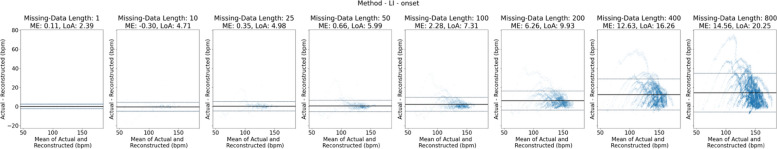
LI - *onset*

**Fig. 22:**
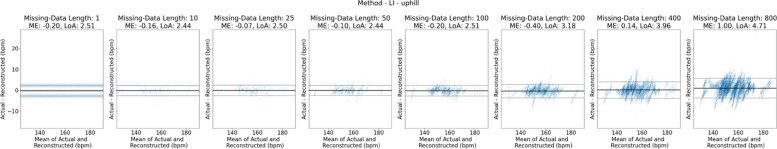
LI - *uphill*

**Fig. 23:**
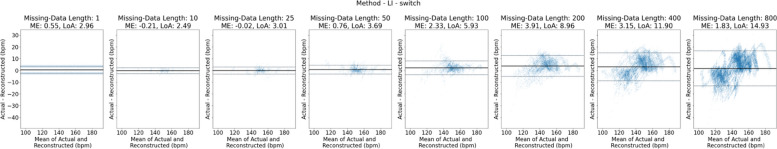
LI - *switch*

**Fig. 24:**
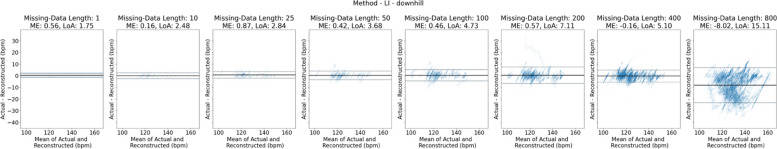
LI - *downhill*

**Fig. 25:**
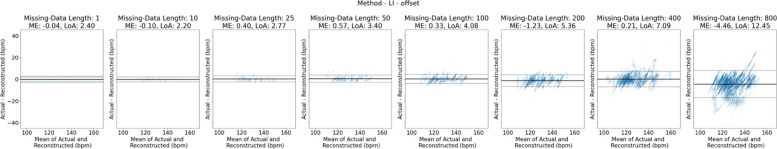
LI - *offset*

## Data Availability

The implementation of the HR model is publicly available on GitHub: https://github.com/tjorvenschnack/HR_Model/tree/2cff8c7c8e8ae6727a9bb2a48b55fcc161471574.

## Appendix A: Results of the Offset Intensity Profile

**Table 4:** Pairwise contrasts between the reconstruction methods, aggregated over all missing-data lengths and intensity profiles (including *offset*)

Contrast	Estimate	CL	t	p	d
HRM - HRMD	0.19	[0.16, 0.22]	14.57	<0.0001	0.54
HRM - LI	0.12	[0.09, 0.15]	9.05	<0.0001	0.33
HRMD - LI	-0.07	[-0.1, -0.04]	-5.51	<0.0001	-0.20

For all rows, the degrees of freedom are 4310.9

**Table 5:** All significant pairwise contrasts between the reconstruction methods, ordered by missing-data lengths and intensity profiles (including *offset*)

Contrast	Intensity profile	Missing-data length	Estimate	CL	t	p	d
HRMD - LI	*downhill*	800	-0.48	[-0.67, -0.29]	-5.78	<0.0001	-1.34
HRMD - LI	*offset*	800	-0.28	[-0.48, -0.09]	-3.41	0.0019	-0.79
HRMD - LI	*switch*	200	-0.39	[-0.58, -0.2]	-4.70	<0.0001	-1.09
HRMD - LI	*switch*	400	-0.47	[-0.67, -0.28]	-5.70	<0.0001	-1.33
HRMD - LI	*switch*	800	-0.55	[-0.74, -0.35]	-6.59	<0.0001	-1.53
HRMD - LI	*onset*	200	-0.31	[-0.51, -0.12]	-3.79	0.0004	-0.88
HRMD - LI	*onset*	400	-0.81	[-1.01, -0.62]	-9.78	<0.0001	-2.27
HRMD - LI	*onset*	800	-1.02	[-1.21, -0.82]	-12.27	<0.0001	-2.85
HRMD - LI	*uphill*	200	0.22	[0.03, 0.42]	2.70	0.0189	0.63
HRMD - LI	*uphill*	400	0.25	[0.06, 0.45]	3.02	0.0072	0.70
HRMD - LI	*uphill*	800	0.27	[0.07, 0.46]	3.21	0.0038	0.75
HRM - HRMD	*downhill*	100	0.24	[0.04, 0.43]	2.88	0.0111	0.67
HRM - HRMD	*downhill*	200	0.23	[0.03, 0.42]	2.71	0.0184	0.63
HRM - HRMD	*downhill*	400	0.25	[0.06, 0.45]	3.06	0.0062	0.71
HRM - HRMD	*offset*	50	0.24	[0.04, 0.43]	2.84	0.0126	0.66
HRM - HRMD	*offset*	100	0.34	[0.14, 0.53]	4.06	0.0001	0.94
HRM - HRMD	*offset*	200	0.38	[0.18, 0.57]	4.56	<0.0001	1.06
HRM - HRMD	*offset*	400	0.37	[0.17, 0.56]	4.43	<0.0001	1.03
HRM - HRMD	*switch*	25	0.23	[0.03, 0.42]	2.73	0.0172	0.64
HRM - HRMD	*switch*	50	0.26	[0.07, 0.46]	3.15	0.0046	0.73
HRM - HRMD	*onset*	10	0.28	[0.09, 0.48]	3.38	0.0021	0.79
HRM - HRMD	*onset*	25	0.35	[0.16, 0.55]	4.25	<0.0001	0.99
HRM - HRMD	*onset*	50	0.37	[0.17, 0.56]	4.40	<0.0001	1.02
HRM - HRMD	*onset*	100	0.41	[0.21, 0.6]	4.92	<0.0001	1.14
HRM - HRMD	*onset*	200	0.21	[0.01, 0.4]	2.48	0.0356	0.58
HRM - HRMD	*uphill*	100	0.35	[0.16, 0.55]	4.22	<0.0001	0.98
HRM - HRMD	*uphill*	200	0.29	[0.1, 0.49]	3.53	0.0012	0.82
HRM - HRMD	*uphill*	400	0.25	[0.05, 0.44]	2.98	0.0081	0.69
HRM - LI	*downhill*	100	0.21	[0.01, 0.4]	2.49	0.0338	0.58
HRM - LI	*downhill*	200	0.26	[0.07, 0.46]	3.16	0.0045	0.73
HRM - LI	*downhill*	400	0.39	[0.19, 0.58]	4.65	<0.0001	1.08
HRM - LI	*downhill*	800	-0.37	[-0.57, -0.18]	-4.46	<0.0001	-1.04
HRM - LI	*offset*	50	0.24	[0.05, 0.44]	2.91	0.0103	0.68
HRM - LI	*offset*	100	0.32	[0.12, 0.51]	3.83	0.0004	0.89
HRM - LI	*offset*	200	0.46	[0.27, 0.66]	5.59	<0.0001	1.30
HRM - LI	*offset*	400	0.28	[0.09, 0.48]	3.42	0.0018	0.79
HRM - LI	*switch*	25	0.27	[0.07, 0.46]	3.20	0.0039	0.74
HRM - LI	*switch*	50	0.32	[0.12, 0.51]	3.82	0.0004	0.89
HRM - LI	*switch*	200	-0.20	[-0.4, -0.01]	-2.42	0.0408	-0.56
HRM - LI	*switch*	400	-0.28	[-0.47, -0.08]	-3.37	0.0022	-0.78
HRM - LI	*switch*	800	-0.37	[-0.57, -0.18]	-4.51	<0.0001	-1.05
HRM - LI	*onset*	10	0.29	[0.09, 0.48]	3.47	0.0015	0.81
HRM - LI	*onset*	25	0.38	[0.19, 0.58]	4.59	<0.0001	1.07
HRM - LI	*onset*	50	0.42	[0.23, 0.62]	5.10	<0.0001	1.19
HRM - LI	*onset*	100	0.40	[0.2, 0.59]	4.80	<0.0001	1.12
HRM - LI	*onset*	400	-0.68	[-0.87, -0.48]	-8.17	<0.0001	-1.90
HRM - LI	*onset*	800	-0.86	[-1.05, -0.66]	-10.30	<0.0001	-2.39
HRM - LI	*uphill*	50	0.26	[0.06, 0.45]	3.10	0.0056	0.72
HRM - LI	*uphill*	100	0.45	[0.26, 0.65]	5.45	<0.0001	1.27
HRM - LI	*uphill*	200	0.52	[0.32, 0.71]	6.23	<0.0001	1.45
HRM - LI	*uphill*	400	0.50	[0.3, 0.69]	6.00	<0.0001	1.40
HRM - LI	*uphill*	800	0.45	[0.26, 0.65]	5.44	<0.0001	1.27

For all rows, the degrees of freedom are 4310

**Table 6:** Mean error (± SD) between actual and reconstructed HR over all samples for the *offset* intensity profile

Intensity profile	Missing-data length	HRM	HRMD	LI
*offset*	1	-0.05 ± 1.59 (2.62)	-0.05 ± 1.59 (2.62)	-0.04 ± 1.46 (2.40)
*offset*	10	-0.65 ± 1.69 (2.79)	-0.19 ± 1.36 (2.24)	-0.10 ± 1.34 (2.20)
*offset*	25	-0.35 ± 1.81 (2.97)	0.11 ± 1.60 (2.63)	0.40 ± 1.68 (2.77)
*offset*	50	-1.38 ± 3.12 (5.12)	-0.28 ± 2.04 (3.35)	0.57 ± 2.07 (3.40)
*offset*	100	-2.73 ± 3.58 (5.89)	-0.91 ± 2.16 (3.55)	0.33 ± 2.48 (4.08)
*offset*	200	-5.27 ± 4.80 (7.90)	-2.43 ± 3.17 (5.21)	-1.23 ± 3.26 (5.36)
*offset*	400	-3.71 ± 5.47 (9.00)	-0.62 ± 3.85 (6.34)	0.21 ± 4.31 (7.09)
*offset*	800	-2.24 ± 6.70 (11.02)	0.47 ± 5.39 (8.87)	-4.46 ± 7.57 (12.45)

Furthermore, the 90% LoA are stated in brackets

## Appendix B: Linear Mixed-Effects Model Diagnostics

Model Specification:

$$\begin{aligned}&log(1 + RMSE) \sim C(ReconstructionMethod) * C(MissingDataLength) *\\ &C(IntensityProfile) + (1 | Participant) \end{aligned}$$

## Appendix C: Results of All Longitudinal Imputation Methods

**Table 7:** All significant pairwise contrasts between LI and the other longitudinal imputation methods, aggregated over all missing-data lengths and intensity profiles

Contrast	Estimate	CL	t	p	d
cubic_spline - LI	0.35	[0.30, 0.40]	18.99	<0.0001	0.70
ffill - LI	0.39	[0.34, 0.44]	21.14	<0.0001	0.78
LI - mean_carried_forward	-0.41	[-0.47, -0.36]	-22.39	<0.0001	-0.82
LI - nearest_neighbor	-0.17	[-0.23, -0.12]	-9.36	<0.0001	-0.34
LI - pchip	-0.07	[-0.13, -0.02]	-3.90	<0.0001	-0.14
LI - trend_carried_forward	-0.76	[-0.82, -0.71]	-41.44	<0.0001	-1.52

**Table 8:** All significant pairwise contrasts between LI and the other longitudinal interpolation methods, ordered by missing-data lengths and intensity profiles

Contrast	Intensity profile	Missing-data length	Estimate	CL	t	p	d
LI - trend_carried_forward	*downhill*	50	-0.54	[-0.89, -0.2]	-4.66	<0.0001	-1.08
LI - trend_carried_forward	*downhill*	100	-0.77	[-1.11, -0.42]	-6.59	<0.0001	-1.53
LI - trend_carried_forward	*downhill*	200	-1.49	[-1.83, -1.14]	-12.75	<0.0001	-2.96
LI - trend_carried_forward	*downhill*	400	-2.10	[-2.44, -1.76]	-18.02	<0.0001	-4.19
LI - mean_carried_forward	*downhill*	800	-0.51	[-0.85, -0.17]	-4.38	<0.0001	-1.02
LI - trend_carried_forward	*downhill*	800	-1.38	[-1.73, -1.04]	-11.87	<0.0001	-2.76
LI - mean_carried_forward	*switch*	25	-0.44	[-0.79, -0.1]	-3.80	<0.0001	-0.88
LI - mean_carried_forward	*switch*	50	-0.56	[-0.9, -0.22]	-4.82	<0.0001	-1.12
LI - trend_carried_forward	*switch*	50	-0.58	[-0.92, -0.24]	-4.98	<0.0001	-1.16
LI - mean_carried_forward	*switch*	100	-0.57	[-0.91, -0.22]	-4.88	<0.0001	-1.13
LI - nearest_neighbor	*switch*	100	-0.43	[-0.77, -0.08]	-3.65	<0.0001	-0.85
LI - trend_carried_forward	*switch*	100	-0.64	[-0.98, -0.3]	-5.50	<0.0001	-1.28
LI - mean_carried_forward	*switch*	200	-0.62	[-0.96, -0.27]	-5.30	<0.0001	-1.23
LI - nearest_neighbor	*switch*	200	-0.35	[-0.7, -0.01]	-3.04	0.0400	-0.71
LI - trend_carried_forward	*switch*	200	-0.67	[-1.01, -0.33]	-5.75	<0.0001	-1.34
LI - mean_carried_forward	*switch*	400	-0.79	[-1.13, -0.45]	-6.78	<0.0001	-1.58
LI - trend_carried_forward	*switch*	400	-0.81	[-1.16, -0.47]	-6.97	<0.0001	-1.62
LI - mean_carried_forward	*switch*	800	-0.77	[-1.11, -0.42]	-6.59	<0.0001	-1.53
LI - trend_carried_forward	*switch*	800	-1.31	[-1.65, -0.96]	-11.22	<0.0001	-2.61
LI - mean_carried_forward	*onset*	1	-0.54	[-0.89, -0.2]	-4.67	<0.0001	-1.09
LI - mean_carried_forward	*onset*	10	-0.74	[-1.09, -0.4]	-6.39	<0.0001	-1.49
LI - mean_carried_forward	*onset*	25	-1.02	[-1.37, -0.68]	-8.77	<0.0001	-2.04
LI - nearest_neighbor	*onset*	25	-0.42	[-0.77, -0.08]	-3.63	0.0100	-0.84
LI - trend_carried_forward	*onset*	25	-0.59	[-0.93, -0.24]	-5.05	<0.0001	-1.17
LI - mean_carried_forward	*onset*	50	-1.34	[-1.68, -1]	-11.51	<0.0001	-2.68
LI - nearest_neighbor	*onset*	50	-0.68	[-1.03, -0.34]	-5.88	<0.0001	-1.37
LI - trend_carried_forward	*onset*	50	-0.87	[-1.22, -0.53]	-7.50	<0.0001	-1.74
LI - mean_carried_forward	*onset*	100	-1.59	[-1.93, -1.24]	-13.63	<0.0001	-3.17
LI - nearest_neighbor	*onset*	100	-0.82	[-1.17, -0.48]	-7.05	<0.0001	-1.64
LI - trend_carried_forward	*onset*	100	-1.11	[-1.45, -0.76]	-9.50	<0.0001	-2.21
LI - mean_carried_forward	*onset*	200	-1.32	[-1.67, -0.98]	-11.34	<0.0001	-2.64
LI - nearest_neighbor	*onset*	200	-0.55	[-0.9, -0.21]	-4.76	<0.0001	-1.11
LI - trend_carried_forward	*onset*	200	-1.08	[-1.42, -0.73]	-9.24	<0.0001	-2.15
LI - mean_carried_forward	*onset*	400	-0.85	[-1.19, -0.51]	-7.29	<0.0001	-1.69
LI - trend_carried_forward	*onset*	400	-1.19	[-1.53, -0.84]	-10.18	<0.0001	-2.37
LI - mean_carried_forward	*onset*	800	-0.75	[-1.09, -0.41]	-6.43	<0.0001	-1.49
LI - trend_carried_forward	*onset*	800	-1.76	[-2.1, -1.41]	-15.07	<0.0001	-3.50
LI - trend_carried_forward	*uphill*	50	-0.45	[-0.79, -0.1]	-3.83	<0.0001	-0.89
LI - trend_carried_forward	*uphill*	100	-0.73	[-1.08, -0.39]	-6.30	<0.0001	-1.46
LI - mean_carried_forward	*uphill*	200	-0.41	[-0.75, -0.06]	-3.50	0.0100	-0.81
LI - trend_carried_forward	*uphill*	200	-1.03	[-1.37, -0.69]	-8.85	<0.0001	-2.06
LI - mean_carried_forward	*uphill*	400	-0.41	[-0.75, -0.06]	-3.48	0.0100	-0.81
LI - trend_carried_forward	*uphill*	400	-1.57	[-1.92, -1.23]	-13.49	<0.0001	-3.14
LI - mean_carried_forward	*uphill*	800	-0.43	[-0.78, -0.09]	-3.71	<0.0001	-0.86
LI - trend_carried_forward	*uphill*	800	-1.97	[-2.31, -1.62]	-16.88	<0.0001	-3.92
cubic_spline - LI	*downhill*	200	0.82	[0.48, 1.17]	7.07	<0.0001	1.64
cubic_spline - LI	*downhill*	400	1.12	[0.77, 1.46]	9.57	<0.0001	2.22
cubic_spline - LI	*downhill*	800	0.80	[0.46, 1.15]	6.90	<0.0001	1.61
ffill - LI	*downhill*	800	0.48	[0.14, 0.83]	4.16	<0.0001	0.97
ffill - LI	*switch*	50	0.52	[0.17, 0.86]	4.44	<0.0001	1.03
ffill - LI	*switch*	100	0.59	[0.24, 0.93]	5.04	<0.0001	1.17
ffill - LI	*switch*	200	0.61	[0.27, 0.95]	5.24	<0.0001	1.22
cubic_spline - LI	*switch*	400	0.40	[0.05, 0.74]	3.42	0.0100	0.79
ffill - LI	*switch*	400	0.79	[0.45, 1.13]	6.78	<0.0001	1.58
cubic_spline - LI	*switch*	800	0.70	[0.36, 1.05]	6.04	<0.0001	1.40
ffill - LI	*switch*	800	0.77	[0.43, 1.12]	6.62	<0.0001	1.54
ffill - LI	*onset*	10	0.50	[0.16, 0.84]	4.29	<0.0001	1.00
ffill - LI	*onset*	25	0.83	[0.49, 1.18]	7.14	<0.0001	1.66
ffill - LI	*onset*	50	1.20	[0.86, 1.55]	10.34	<0.0001	2.40
ffill - LI	*onset*	100	1.50	[1.16, 1.84]	12.87	<0.0001	2.99
ffill - LI	*onset*	200	1.26	[0.91, 1.6]	10.79	<0.0001	2.51
ffill - LI	*onset*	400	0.79	[0.45, 1.14]	6.81	<0.0001	1.58
ffill - LI	*onset*	800	0.70	[0.35, 1.04]	5.97	<0.0001	1.39
cubic_spline - LI	*uphill*	100	0.39	[0.05, 0.74]	3.37	0.0100	0.78
ffill - LI	*uphill*	100	0.35	[0.01, 0.7]	3.03	0.0400	0.70
cubic_spline - LI	*uphill*	200	0.48	[0.14, 0.82]	4.11	<0.0001	0.96
ffill - LI	*uphill*	200	0.44	[0.09, 0.78]	3.75	<0.0001	0.87
cubic_spline - LI	*uphill*	400	0.72	[0.38, 1.07]	6.19	<0.0001	1.44
ffill - LI	*uphill*	400	0.43	[0.09, 0.78]	3.72	<0.0001	0.87
cubic_spline - LI	*uphill*	800	1.18	[0.83, 1.52]	10.09	<0.0001	2.35
ffill - LI	*uphill*	800	0.44	[0.09, 0.78]	3.74	<0.0001	0.87

## Appendix D: Intra-Class Correlation Coefficient and Coefficient of Variation

**Table 9:** ICC(2,1) and CV for the RMSE across the repeated bouts of each reconstruction method

Reconstruction Method	F	ICC(2,1)	*p*	CV [%]
HRMD	5.0240	0.2697	0.0081	41.9387
LI	15.2501	0.3927	<0.0001	54.3275

The corresponding F-statistic an *p*-value are stated alongside the ICC values. All participants performed at least three bouts, therefore the metrics were determined for the first three bouts

## Appendix E: Statistical Results Details

**Table 10:** Significance of fixed effects and their interactions

	Sum Sq	Mean Sq	NumDF	DenDF	F value	Pr($\boldsymbol{>}$F)
IntensityProfile	76.90690	25.63563	3	3446.853	196.27099	0
ReconstructionMethod	20.73656	10.36828	2	3446.853	79.38140	0
MissingDataLength	581.08671	83.01239	7	3446.853	635.55764	0
IntensityProfile:ReconstructionMethod	14.72464	2.45411	6	3446.853	18.78908	0
IntensityProfile:MissingDataLength	38.04407	1.81162	21	3446.853	13.87011	0
ReconstructionMethod:MissingDataLength	25.99739	1.85696	14	3446.853	14.21719	0
IntensityProfile:ReconstructionMethod:MissingDataLength	39.17156	0.93266	42	3446.853	7.14058	0

Type III analysis of variance table with Satterthwaite’s method

## Appendix F: Bland Altman Plots

Bland Altman plots for all reconstructed HR values, ordered by reconstruction method, intensity profile and missing-data length. ME and LoA are reported in the plot title and represented by the solid line and the dotted lines, respectively.

## Abbreviations

bpm: Beats per minute
BRITS: Bidirectional Recurrent Imputation for Time Series
CL: Confidence limits
CV: Coefficient of variation
GNSS: Global Navigation Satellite System
HR: Heart rate
HRM: Heart rate model reconstruction approach
HRMD: Heart rate model reconstruction approach with drift correction
ICC: Intra-class correlation coefficient
LI: Linear interpolation
LoA: 90% limits of agreement
MDES: Minimum detectable effect size
ME: Mean error
RE3: Running energy-expenditure estimation
RMSE: Root mean square error
SD: Standard deviation
